# Exploring the Role of the Head Rotation Sit-Up Test in Identifying Epley-Responsive, Non-Classical Presentations of BPPV

**DOI:** 10.3390/medicina62010217

**Published:** 2026-01-20

**Authors:** Ryo Yoneima, Kenji Nishio, Hiromasa Kawashima, Sota Sakamoto, Tomohiro Nakamura, Akihiro Sawa, Ayaka Kakiwaki, Hiroyuki Taguchi, Satoshi Senzaki, Nobushiro Nishimura, Hidetoshi Matsuoka, Shiro Ono, Masaki Matsubara, Noritaka Yada, Kiyomi Yoshimoto

**Affiliations:** 1 Department of General Medicine, Nara Medical University Hospital, Kashihara 634-8522, Nara, Japan; ryo_1122@naramed-u.ac.jp (R.Y.); kawashima.h@naramed-u.ac.jp (H.K.); s.sakamoto@naramed-u.ac.jp (S.S.); t-nakamura@naramed-u.ac.jp (T.N.); akihirosawa@naramed-u.ac.jp (A.S.); a.kakiwaki@naramed-u.ac.jp (A.K.); taguchi4161@gmail.com (H.T.); sen.senzaki5008.golf@gmail.com (S.S.); nobushiro.n@gmail.com (N.N.); hmatsu1227@naramed-u.ac.jp (H.M.); shiroono0207@gmail.com (S.O.); m.matsubara@naramed-u.ac.jp (M.M.); n-yada@naramed-u.ac.jp (N.Y.); kym@naramed-u.ac.jp (K.Y.); 2 Department of General Medicine, Uda City Hospital, Uda 633-0298, Nara, Japan

**Keywords:** BPPV, Dix–Hallpike test, positioning nystagmus test, Epley maneuver, Head Rotation Sit-up Test

## Abstract

*Background and Objectives*: The Dix–Hallpike test (DHT) is the standard diagnostic maneuver for posterior semicircular canal benign paroxysmal positional vertigo (BPPV). However, some patients present with positional symptoms compatible with BPPV yet show no observable nystagmus on the DHT. We introduced the Head Rotation Sit-up Test (HRST) as a symptom-based maneuver and hypothesized that it would identify DHT-negative patients who nonetheless benefit from canalith repositioning. This study aimed to explore the potential role of the HRST. It was introduced as a complementary, symptom-based maneuver for evaluating non-classical vertigo presentations. *Materials and Methods*: We retrospectively reviewed patients clinically suspected of having BPPV by the attending physicians at Nara Medical University Hospital (August 2018–July 2022). All underwent both the DHT and HRST; those positive on either test received the Epley maneuver and were included. Patients were categorized as Group 1: DHT(−)/HRST(+), and Group 2: DHT(+), irrespective of the HRST, for the purpose of comparing patients who were positive on the HRST with those positive on the DHT. Post-treatment symptom severity was assessed on a 0–10 scale, with the patient’s most severe pre-treatment symptom defined as 10, and responder rates (post-Epley score < 5) were compared. *Results*: Among 179 patients with suspected BPPV, 80 were test-positive and were treated with the Epley maneuver. Group 1 comprised 31 patients, who more commonly reported non-rotational symptoms such as floating or unsteadiness, whereas rotational vertigo predominated in Group 2 (*n* = 49). Median post-Epley scores were significantly lower after treatment in both groups (Group 1: 2 [IQR 0–3]; Group 2: 3 [IQR 0–5]). Group 1 demonstrated a higher responder rate than Group 2 (90.3% vs. 65.3%, *p* = 0.016). Within Group 2, outcomes did not differ significantly between DHT(+)/HRST(+) and DHT(+)/HRST(−) subgroups. *Conclusions*: The HRST identified a clinically relevant subgroup of DHT-negative patients who nevertheless responded favorably to the Epley maneuver. Incorporating the HRST alongside the DHT may expand the diagnostic reach for BPPV, particularly among patients with non-classical symptoms or without observable nystagmus. These findings support the HRST as a useful complementary maneuver for detecting Epley responsive, symptom based positional vertigo.

## 1. Introduction

Benign paroxysmal positional vertigo (BPPV) is the most common peripheral vestibular disorder and is characterized by brief episodes of vertigo triggered by specific head positions or positional changes. BPPV accounts for approximately one-quarter of all vertigo cases in clinical practice, and when probable cases are included, it may represent over 40% of vertigo presentations [1,2]. The widely accepted mechanism of BPPV involves otoconia dislodged from the utricle entering a semicircular canal and stimulating the cupula or endolymphatic flow, thereby provoking vertigo [3,4]. Posterior canal BPPV (PC-BPPV) represents the majority of BPPV cases [5,6]. Because the Dix–Hallpike test (DHT) most reliably provokes posterior canal-related otoconial movement and the resulting characteristic positional nystagmus, it has been established as the standard diagnostic maneuver for BPPV [7]. During the DHT, the patient’s head is turned 45° to one side and rapidly brought to a supine head-hanging position, where the presence of characteristic torsional up-beating nystagmus confirms the diagnosis. Once PC-BPPV is identified, the Epley maneuver serves as an effective canalith repositioning treatment, with a reported number needed to treat (NNT) of approximately 3 [8].

Clinically, BPPV typically presents as positionally triggered vertigo, such as when rolling over in bed, bending forward, or looking upward [9]. However, a subset of patients presents with characteristic positional symptoms of BPPV. Despite this, standard positional tests such as the DHT and head roll tests can yield negative results without observable nystagmus. During routine clinical evaluations of such patients, our attention was drawn to the fact that positional symptoms rather than nystagmus were sometimes provoked during the transition from the head-hanging position (with 45° head rotation) back to sitting upright. Upon further clinical observation of this phenomenon, we noted that certain patients demonstrated a distinct side-to-side difference in the intensity of positional symptoms such as vertigo or unsteadiness during this sit-up transition, even when the DHT yielded negative results. Based on this observation, we introduced this maneuver as the Head Rotation Sit-up Test (HRST), a symptom-based adjunct to conventional positional testing. Regardless of the presence or absence of nystagmus during HRST, when a clear difference in symptom intensity was observed between the two directions of head rotation, we performed the Epley maneuver on the side with more pronounced symptoms and found that many of these patients experienced substantial symptomatic improvement. Thus, we hypothesized that the HRST would provide insight into Epley-responsive, non-classical presentations within the spectrum of PC-BPPV that are not accompanied by observable nystagmus.

Accordingly, the present study was designed to explore the potential role of the HRST in the clinical evaluation of such patients.

## 2. Materials and Methods

### 2.1. Procedures

#### 2.1.1. Dix–Hallpike Test (DHT)

The DHT was performed according to established guidelines [3]. Eye movements were recorded using video-oculography with a goggle-mounted infrared camera system, and nystagmus was assessed from the recordings. All positional tests were performed by attending physicians experienced in vestibular examination.

The DHT was considered positive when characteristic torsional up-beating nystagmus was observed during the maneuver.

#### 2.1.2. Head Rotation Sit-Up Test (HRST)

The HRST was performed immediately after the DHT. While the patient was in the supine head-hanging position, the head was rotated 45° to one side. The patient was then brought rapidly to the sitting position while maintaining the same head rotation, but with cervical extension released during sit-up movement.

Unlike the DHT, visible nystagmus was not required for a positive HRST result.

During the HRST, patients experienced either rotational vertigo or a brief floating sensation. Although their chief presenting complaints varied, the symptoms that were during HRST were limited to these two types. Thus, the HRST focused on a narrow range of vestibular sensations rather than nonspecific discomfort. To evaluate asymmetry, patients were asked to rate the intensity of the same symptom on both sides using a 0–10 numerical scale. The more symptomatic side was designated as 10. The opposite side was then rated relative to this reference. If the contralateral rating was less than 5, indicating that symptom intensity on that side was perceived as less than half of that on the ipsilateral side, the result was considered HRST-positive. This approach allowed a standardized, patient-reported comparison of symptom asymmetry between sides, regardless of the specific symptom present.

This threshold (<5) was determined based on early clinical experience with the HRST. It was chosen to prioritize diagnostic clarity and lateralization reliability, and to minimize ambiguity in symptom asymmetry. When the HRST result was positive, the more symptomatic side was regarded as the affected side.

#### 2.1.3. Epley Maneuver

The Epley maneuver was performed on the side indicated by a positive DHT or HRST result. Although the maneuver is well established and widely described in the literature [3,10], the standard protocol at our institution was to perform three consecutive repetitions of the maneuver in all patients. Each position of the maneuver was maintained for approximately 90 s. In some patients, completion of all three repetitions was not possible because of discomfort, nausea, or intolerance during the procedure. In such cases, the maneuver was discontinued at a point deemed clinically appropriate.

### 2.2. Patient Selection

We retrospectively reviewed patients who visited the Department of General Medicine, Nara Medical University Hospital, between August 2018 and July 2022. Eligible patients were those who were clinically suspected of having BPPV by the attending physician. This clinical suspicion was based primarily on a characteristic history of sudden-onset vertigo that was triggered reproducibly by specific head movements or positional changes. The vertigo was typically rotational in nature and lasted from several seconds to a few minutes. Patients with accompanying symptoms suggestive of central or non-vestibular causes, including hearing loss, tinnitus, focal neurological deficits, or headache were excluded from the study population.

In addition to these classical presentations, we also included patients who reported symptoms such as brief sensations of swaying, a floating or unsteady feeling, a sense of not being firmly grounded while walking, or a tendency to lean or veer to one side when those symptoms were clearly provoked or exacerbated by head or body movement.

Furthermore, patients presenting with sudden-onset nausea or vomiting were also considered for BPPV evaluation when no abnormalities were found in blood pressure, gastrointestinal motility, focal neurological examination, or headache assessment, particularly if symptom onset appeared temporally related to head movement or positional change.

When any of these clinical features were present, BPPV was routinely suspected, and both the DHT and the HRST were performed as part of the standard clinical assessment at our department. Patients who demonstrated a positive result on either positional test subsequently underwent the Epley maneuver. The present study included only those patients who received the Epley maneuver based on a positive result in at least one of these positional tests.

### 2.3. Data Collection

Age, sex, duration from symptom onset to presentation, whether symptoms first occurred upon awakening, the chief presenting complaint (defined as the single symptom the patient identified as most bothersome), findings from positional tests (DHT and HRST), and treatment responses after the Epley maneuver were obtained from the electronic medical records.

### 2.4. Group Classification

Based on the results of the positional tests, patients were classified into two groups to compare patients who were HRST-positive with those who were DHT-positive. Group 1 consisted of those with DHT(−)/HRST(+), representing patients in whom suspected BPPV was not identified by the conventional DHT but was detected by the HRST. Group 2 consisted of those with DHT(+), regardless of HRST findings, representing patients who were identifiable using the standard DHT and diagnosed as having PC-BPPV. Group 2 was further subdivided according to HRST results into a DHT(+)/HRST(+) subgroup and a DHT(+)/HRST(−) subgroup for further comparative analysis.

### 2.5. Outcome Assessment

Treatment response to the Epley maneuver was quantified using a patient-reported numerical rating scale (0–10). Each patient was instructed to score the severity of their chief complaint as 10 before the Epley maneuver, including rotational vertigo, floating sensation, or unsteadiness. After the Epley maneuver, patients were asked to stand and walk a short distance, as symptoms typically become apparent during these movements. Based on the severity of the same chief complaint experienced during and after these postural changes, patients were then asked to rate their symptoms again on the same 0–10 numerical scale. This post-treatment rating was defined as the post-Epley symptom score. The primary outcome was the therapeutic effect of the Epley maneuver, assessed in two ways: (1) the degree of symptom improvement within each group, and (2) comparison of post-treatment symptom scores between Groups 1 and 2.

For categorical analysis, patients with post-Epley symptom scores < 5 were classified as responders (effective group), whereas those with scores ≥ 5 were classified as non-responders (ineffective group). This cutoff value was determined based on our clinical experience and retrospective consensus among the investigators. The proportion of responders was compared between the two groups.

As a secondary analysis, we further compared post-treatment symptom scores and response rates between the DHT(+)/HRST(+) and DHT(+)/HRST(−) subgroups within Group 2 to evaluate the differential impact of HRST positivity among patients already identified by the DHT.

### 2.6. Statistical Analysis

Categorical variables were compared using Fisher’s exact test and continuous variables were compared using the Mann–Whitney U test. Normality was assessed using the Shapiro–Wilk test. The 95% confidence intervals for both responder proportions and their difference were calculated using the Wilson score method for proportions and the Newcombe (Wilson–Wilson) method for the difference in proportions. Analyses were performed using EZR version 1.68 [11]. Statistical significance was set at *p* < 0.05.

### 2.7. Ethical Statement

This study was approved by the Nara Medical University Ethics Committee (approval number: 2110). The study was performed in accordance with the tenets of the Declaration of Helsinki and the Good Clinical Practice guidelines.

## 3. Results

### 3.1. Study Population

A total of 179 patients with clinical suspicion of BPPV underwent positional testing using the DHT and the HRST. Among these, 80 patients demonstrated a positive finding on at least one of the positional tests and subsequently received the Epley maneuver. Of these 80 patients, 31 were classified in Group 1 (DHT(−)/HRST(+)), and the remaining 49 were classified in Group 2 (DHT(+), regardless of HRST results) as shown in Table 1. Within Group 2, 39 patients were positive for both the DHT and the HRST, whereas 10 patients demonstrated DHT(+)/HRST(−) findings. Age, sex distribution, and the median duration from symptom onset to presentation did not differ significantly between Group 1 and Group 2. The proportion of patients whose symptoms began upon awakening also did not differ significantly between groups.

In contrast, several chief presenting symptoms showed statistically significant differences between the two groups. Rotational vertigo, the classical hallmark of PC-BPPV, was significantly more common in Group 2 (30/49) than in Group 1 (4/31) (*p* < 0.001). Conversely, non-rotational symptoms were significantly more frequent in Group 1. Floating sensation was more common in Group 1 than in Group 2 (11/31 vs. 4/49; *p* = 0.003), as was unsteadiness or imbalance (10/31 vs. 6/49; *p* = 0.044).

Other symptoms, including swaying or tilting sensation, a feeling of not being firmly grounded while walking, veering to one side, and nausea did not show significant differences between groups.

These findings indicate that Group 1 (DHT(−)/HRST(+)) more frequently presented with non-rotational, less classical vertigo symptoms, whereas Group 2 (DHT(+)) more frequently exhibited typical rotational vertigo. Notably, although patients who reported rotational vertigo often also endorsed additional sensations such as floating or unsteadiness, the chief complaints in most of these cases remained spinning vertigo. In contrast, among patients who did not report rotational vertigo, the chief complaints were more heterogeneous, commonly including floating sensation, unsteadiness, or other non-rotational symptoms.

### 3.2. Post-Epley Symptom Score

Post-treatment symptom scores showed significant improvement within both groups. Using a numerical rating scale (0–10), with pre-treatment symptom severity defined as

### 3.3. Responder Analysis (Table 2 and Figure 1)

When treatment response was evaluated categorically using a post-Epley symptom score < 5 as the threshold for clinical effectiveness, Group 1 demonstrated a significantly higher responder rate than Group 2. As shown in Table 2, responders comprised 28 of 31 patients (90.3% [95% CI: 74.2% to 97.4%]) in Group 1, compared with 32 of 49 patients (65.3% [95% CI: 50.4% to 78.3%]) in Group 2 (Fisher’s exact test, *p* = 0.016; odds ratio [OR] = 4.96 [95% CI: 1.31–18.73]). The difference in proportions was 25% [95% CI: 5.8% to 40.4%], as calculated using the Newcombe (Wilson–Wilson) method. Accordingly, patients identified through the HRST showed a significantly higher likelihood of clinically meaningful symptomatic improvement following the Epley maneuver.

**Figure 1:**
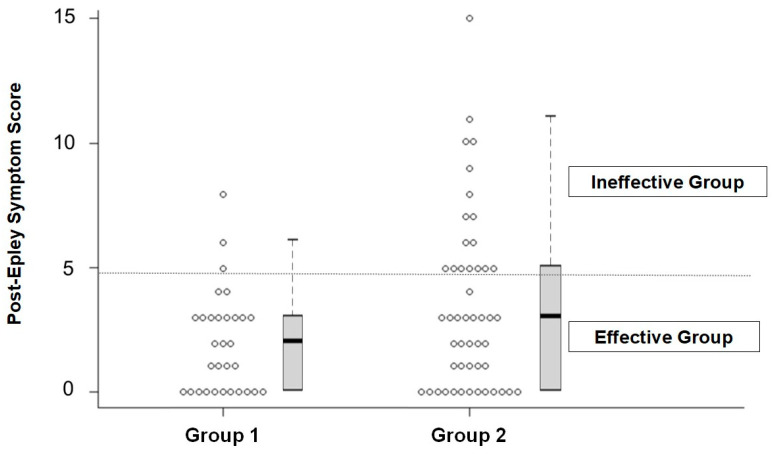
Individual post-Epley symptom scores on a 0–10 scale are shown for each group. (Group 1, *n* = 31; Group 2, *n* = 49). Overlaid box-and-whisker plots represent the distribution of post-treatment scores, with the boxes indicating the interquartile range (IQR), the bold horizontal line representing the median, and the whiskers extending to the minimum and maximum values within 1.5 × IQR. Patients with post-Epley symptom score < 5 were classified as effective, and those with scores ≥ 5 as ineffective; the cutoff is indicated by the horizontal dotted line. The proportion of patients classified as effective was significantly higher in Group 1 than in Group 2 (*p* = 0.016). 10, the median post-Epley symptom score was 2 (IQR, 0–3) in Group 1 and 3 (IQR, 0–5) in Group 2. Symptom reduction was significant within each group (both *p* < 0.001). Comparison of the two groups revealed no statistically significant difference in post-treatment scores (*p* = 0.095), although Group 1 exhibited numerically lower scores overall.

**Table 2:** Comparison of Efficacy Rates Between Group 1 and Group 2.

	Group 1 (*n* = 31)	Group 2 (*n* = 49)	Total
Effective Group (post-Epley symptom score = 0~4)	28	32	60
Ineffective Group (post-Epley symptom score ≥ 5)	3	17	20
Total	31	49	80
			*p* = 0.016

Fisher’s exact test was used for effectiveness rates to compare the two groups.

### 3.4. Subgroup Analysis Within Group 2 (Figure 2)

**Figure 2:**
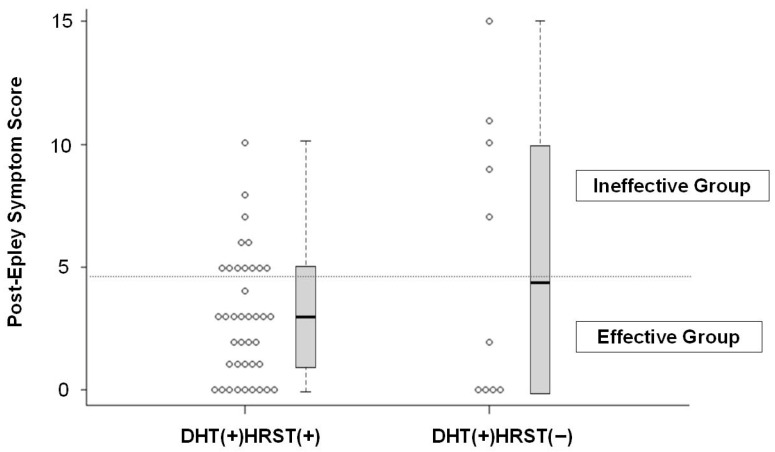
Comparison of post-Epley symptom scores between the DHT(+)/HRST(+) and DHT(+)/HRST(−) subgroups (DHT(+)/HRST(+), *n* = 39; DHT(+)/HRST(−), *n* = 10). Each circle represents an individual patient’s post-Epley symptom score. Patients with post-Epley symptom scores < 5 were classified as effective, and those with scores ≥ 5 as ineffective; the cutoff is indicated by the horizontal dotted line. The proportion of patients classified as effective was 69.2% (27/39) in the DHT(+)/HRST(+) subgroup and 50.0% (5/10) in the DHT(+)/HRST(−) subgroup, with no statistically significant difference between the two subgroups (*p* = 0.285).

Within Group 2, further analysis was conducted to compare the DHT(+)/HRST(+) and DHT(+)/HRST(−) subgroups (Figure 2). The DHT(+)/HRST(+) subgroup included 39 patients, with a median post-Epley symptom score of 3 (interquartile range [IQR], 1–5), whereas the DHT(+)/HRST(−) subgroup consisted of 10 patients, with a median post-Epley symptom score of 4.5 (IQR, 0–9.75). There was no significant difference in post-Epley symptom scores between the two subgroups (*p* = 0.458). The proportion of treatment responders (post-Epley score <5) was 69.2% (27/39) in the DHT(+)/HRST(+) subgroup and 50.0% (5/10) in the DHT(+)/HRST(−) subgroup, with no statistically significant difference between the two subgroups (*p* = 0.285).

## 4. Discussion

### 4.1. Key Findings

This study demonstrated that the HRST was able to identify, among patients who do not exhibit classical rotational vertigo but present with non-rotational symptoms such as floating sensation or unsteadiness, a subgroup with PC-BPPV–like features whose symptoms subsequently improved after the Epley maneuver. Such cases are often not initially suspected of having BPPV in primary-care settings, and even when evaluated using the DHT, they frequently exhibit no observable nystagmus, rendering them prone to being overlooked in routine clinical practice. The HRST appears valuable for identifying a subgroup of patients who respond well to the Epley maneuver. However, when the DHT is negative, these cases cannot be diagnosed as definite BPPV under current criteria. Therefore, these cases remain classified as suspected or probable BPPV, highlighting the need for further refinement of diagnostic frameworks to accommodate symptom-based maneuvers such as the HRST.

Compared with DHT-positive patients (Group 2), Group 1 was characterized by a significantly higher prevalence of non-rotational symptoms (floating sensation and unsteadiness/imbalance) and a significantly lower prevalence of classical rotational vertigo at presentation (Table 1).

As shown in Table 1, we emphasize the importance of documenting each patient’s chief complaint as described in their own words, recognizing that these expressions, though often similar in nature, can carry important clinical nuances. When such descriptors are reported, including terms like “floating sensation” or “unsteadiness,” clinicians should consider the possibility of BPPV. While these non-rotational symptoms were significantly more common among HRST-only positive cases, it is important to note that some patients with DHT-confirmed BPPV also presented with these symptoms as their primary complaint. This observation highlights the need for clinicians to consider the possibility of BPPV among patients presenting with non-rotatory symptoms in general practice.

### 4.2. Pathophysiological Basis of the HRST

The potential relationship between these non-rotational symptoms and BPPV was initially recognized through our clinical experience, when we encountered patients with PC-BPPV whose classical rotational vertigo resolved after undergoing the Epley maneuver yet who continued to experience floating sensation or unsteadiness. These symptoms were often influenced by head movement or changes in body position. We hypothesized that they may represent mild or residual BPPV, possibly caused by a reduced otoconial load insufficient to provoke typical vertigo. If the HRST can reliably identify a subset of patients with such non-rotational symptoms who nonetheless respond to the Epley maneuver, it may serve as a useful tool for detecting milder forms of BPPV that elude conventional diagnostic criteria.

The HRST is performed with the head rotated 45 degrees to one side to selectively align the posterior semicircular canal on the ipsilateral side with the direction of upper-body motion during the sit-up transition. This orientation facilitates endolymphatic flow and otoconial movement in the posterior canal on the side of rotation. For instance, when the head is turned to the right, the right posterior canal becomes aligned with the plane of movement, making it more responsive to positional changes, while the contralateral posterior canal remains relatively inactive due to its orthogonal orientation. Consequently, side-to-side differences in symptom provocation during the HRST are more likely to reflect unilateral posterior canal involvement rather than non-vestibular or centrally mediated causes.

A plausible physiological interpretation of the HRST response is that the sit-up transition induces a transient reduction in cerebral and cerebellar perfusion, particularly in individuals with mild autonomic dysregulation or orthostatic susceptibility, thereby lowering the inhibitory control normally exerted by the cerebellum on vestibular input [12,13]. A more specific and clinically relevant interpretation for the DHT(−)/HRST(+) cases is that this transient circulatory change unmasks subtle posterior canal excitation that is too weak to produce observable nystagmus during the DHT but becomes perceptible as a floating sensation or unsteadiness during the sit-up phase.

Such circulatory vulnerability induced by autonomic dysregulation may lower the threshold at which canalith-related signals become clinically perceptible, which could partly explain why autonomic symptoms and BPPV frequently coexist [12].

### 4.3. Relationship to Existing Diagnostic Strategies

Traditional BPPV diagnosis hinges on visualizing canal-specific nystagmus and determining the involved semicircular canal [3,14]. The DHT is optimized to detect PC-BPPV by provoking ampullofugal endolymph movement. However, even with infrared video-oculography, a substantial proportion of clinically suspected cases exhibit no visible nystagmus, reflecting either subtle canalithiasis or limitations inherent to real-world clinical observation [15].

The HRST complements this paradigm by shifting the emphasis from eye-movement–based observation to patient-reported, lateralized symptoms during the sit-up transition. Conceptually, this approach aligns with the notion of subjective BPPV (s-BPPV), in which patients experience vertigo during the DHT despite the absence of visually detectable nystagmus [16]. In s-BPPV, the presumed mechanism is that only a small quantity of otoconial debris or weak endolymphatic flow is generated, which is insufficient to trigger observable nystagmus but still capable of producing vestibular symptoms. Although s-BPPV is defined by symptom provocation during the descent into the head-hanging position, whereas the HRST evaluates symptoms during the return to the sitting position, the two approaches share a key conceptual similarity: both rely primarily on symptom reproduction rather than visible nystagmus. Importantly, canalith repositioning maneuvers have been shown to be effective in s-BPPV, suggesting that symptom-based detection can still reflect clinically meaningful posterior-canal involvement.

In a previous report, 35 of 162 patients with suspected BPPV were classified as s-BPPV, indicating that a substantial subset of BPPV cases shows symptom-only responses without observable nystagmus [16]. This aligns with our findings that a notable number of patients in Group 1 were DHT(−) yet HRST(+), suggesting that symptom-based maneuvers may uncover a group of “subtle” or “hidden” BPPV that conventional nystagmus-based assessments may miss.

To avoid confusion with the HRST, it is worth distinguishing this approach from the so-called Reverse DHT [17]. The Reverse DHT also focuses on the transition from the head-hanging position back to sitting, but its diagnostic value still depends on observing nystagmus in the opposite direction. No study has directly compared the sensitivity of the Reverse DHT to the standard DHT. It also remains unclear whether otoconia more readily provoke nystagmus during descent or ascent. Nevertheless, during the DHT, it is physiologically plausible that lowering the upper body into the head-hanging position produces more reliable ampullofugal endolymph flow in the posterior semicircular canal, which is more excitatory than the ampullopetal flow generated during the return to sitting. Therefore, the standard DHT is presumed to be more effective in eliciting nystagmus than the Reverse DHT. Taken together, because the HRST relies on the return-to-sitting movement, the stimulus applied to the posterior semicircular canal may be reduced, which could account for the lower likelihood of provoking rotational vertigo during the HRST.

### 4.4. Interpretation and Clinical Implications of HRST Positivity

Importantly, the DHT(−)/HRST(+) group showed a significantly greater clinical response to the Epley maneuver compared with DHT-positive patients. The responder threshold (<5) was based on the change in intensity of the same chief complaint rated before and after treatment. Since all baseline scores were fixed at 10 before the Epley maneuver, this cutoff reflects a reduction greater than 50%. When a threshold of <6 was applied, the DHT(−)/HRST(+) group continued to demonstrate a significantly greater clinical response (Fisher’s exact test, *p* = 0.047), supporting the robustness of the findings. Notably, many HRST-positive patients primarily reported non-rotational symptoms, including floating sensation or unsteadiness, rather than classical vertigo. As previously discussed, these symptoms may reflect a milder form of posterior canal involvement, possibly due to a reduced otoconial load. This may explain the higher rate of response to canalith repositioning therapy in this group.

In this regard, HRST positivity may identify a subgroup of patients who are particularly likely to benefit from canalith repositioning, even when classical nystagmus-based tests fail to demonstrate overt posterior-canal involvement. Additionally, the HRST may function not only as a diagnostic adjunct but also as a pragmatic tool for anticipating therapeutic yield among patients with suspected PC-BPPV.

When we further evaluated whether HRST positivity could enhance prognostic stratification within the DHT(+) subgroup, a numerically higher response rate was observed in DHT(+)/HRST(+) patients than in DHT(+)/HRST(−) patients, as shown in Figure 2. However, this trend did not reach statistical significance. One possible explanation is that patients with DHT-confirmed BPPV generally harbor a larger burden of canaliths or more robust canal stimulation. In such cases, the incremental contribution of the HRST, which ability to unmask subtle posterior-canal stimulation may be inherently diminished, thereby attenuating its predictive value for treatment response.

In our department, patients presenting with floating sensation or unsteadiness, which are symptoms often attributed to autonomic dysfunction, nonetheless routinely undergo positional testing. This approach is based on our clinical experience that such symptoms may still represent a BPPV-like condition responsive to the Epley maneuver, despite appearing consistent with autonomic dysfunction. Among 179 such patients, only 80 (44.7%) demonstrated a positive result on either the DHT or HRST, a proportion consistent with the expectation that many cases represented non-vestibular etiologies such as orthostatic intolerance, which may arise from cardiovascular or pulmonary dysfunction, volume depletion, or psychogenic factors. Crucially, however, autonomic dysfunction, including orthostatic intolerance, does not generate lateralized responses during positional testing such as the DHT or the HRST. Lateralized symptom provocation is a hallmark of peripheral vestibular asymmetry and is not expected in dizziness attributable to systemic circulatory instability.

Thus, even among patients who present only with floating sensation or unsteadiness, symptoms often attributed to autonomic dysfunction, clinicians should recognize that an underlying condition responsive to the Epley maneuver may be concealed and consider performing both the DHT and the HRST.

From a practical standpoint, it is noteworthy that 70 of the 80 patients who ultimately received the Epley maneuver were HRST-positive, corresponding to an HRST positivity rate of 87.5% in this treated group. This suggests that, even if the DHT is not routinely performed or nystagmus cannot be reliably observed, the HRST alone has the potential to identify the majority of patients who will benefit from canalith repositioning. At the same time, 10 of 80 Epley-treated patients were DHT(+)/HRST(−), underscoring that the HRST should be viewed as a complementary tool rather than a replacement for the DHT when feasible. From a clinical perspective, the HRST may be particularly useful in primary care or general medical settings where nystagmus cannot always be reliably observed. Incorporating the HRST into routine positional assessment may help identify patients likely to benefit from canalith repositioning therapy, even when classical signs are absent

### 4.5. Limitations

This study has several limitations. First, its retrospective design inherently restricts control over confounding factors and limits the ability to infer causality. Only patients who underwent the Epley maneuver were included; therefore, individuals with DHT(−)/HRST(−) results were not evaluated, and the diagnostic specificity of the HRST could not be determined.

Second, the study was conducted at a single university hospital, where many patients were referred from community clinics. As a result, the study population may have included a relatively low proportion of typical, easily recognizable cases of PC-BPPV, while a larger number of patients presented after a prolonged duration since symptom onset. This referral bias may limit the generalizability of our findings to primary care settings, where earlier and more classical cases are commonly encountered.

Third, although video-oculography was used for nystagmus detection, very subtle eye movements may still have been overlooked. A trained vestibular specialist might have identified additional fine nystagmus that could have influenced the classification of DHT-negative cases [18,19]. In addition, all positional tests were performed by attending physicians experienced in vestibular examination; however, a formal inter-rater reliability assessment was not conducted, which represents an additional methodological limitation.

Fourth, the HRST positivity threshold (10 vs. <5) was based on clinical experience and not formally validated. This cutoff was chosen to improve diagnostic clarity by identifying clearly lateralized and unambiguous symptom responses while avoiding borderline cases. However, the lack of prospective validation limits the generalizability of this criterion, and further studies are required to determine its optimal cutoff value.

Fifth, treatment response was evaluated using a patient-reported numerical symptom scale. This approach reflects clinically meaningful improvement from the patient’s perspective. However, it remains inherently subjective and may be influenced by expectation effects or recall bias. Furthermore, no objective vestibular function tests, such as vestibular evoked myogenic potentials or quantitative canal function assessments, were performed to corroborate the presumed posterior canal involvement.

Sixth, the study lacked blinding of both examiners and patients, and no prospective control group, including sham maneuvers was incorporated. Consequently, the contribution of placebo effects or spontaneous symptom resolution could not be fully excluded, and the predictive value of the HRST may have been overestimated.

Finally, the sample sizes of certain subgroups, particularly in the DHT(+)/HRST(−) group, were relatively small, which may have limited statistical power and increased the risk of type I error. In addition, long-term follow-up was not available, precluding assessment of symptom recurrence or sustained treatment effects.

Taken together, these limitations underscore the need for future prospective, blinded studies with larger sample sizes, objective vestibular assessments, standardized inter-rater reliability assessments, and long-term follow-up in both primary care and emergency settings to more robustly validate the diagnostic and clinical utility of the HRST.

## 5. Conclusions

The HRST identified a subgroup of patients with non-classical positional symptoms who were negative on the DHT yet showed meaningful clinical improvement after the Epley maneuver. Although these cases cannot be classified as definite BPPV under current nystagmus-based criteria, their lateralized symptom responses and favorable treatment outcomes suggest the presence of mild or attenuated posterior semicircular canal involvement, conceptually aligned with subjective BPPV. Clinically, the HRST may serve as a practical and accessible adjunct to standard positional testing, particularly in settings where nystagmus observation is limited. To validate its diagnostic and therapeutic relevance, future studies should employ prospective, blinded designs with objective vestibular assessments and long-term follow-up across broader clinical settings, including primary and emergency care.

## Figures and Tables

**Table 1:** Baseline Characteristics of the Study Participants.

Variable	Group 1 (*n* = 31)	Group 2 (*n* = 49)	*p* Values
**Age, years (range)**	63 (17–85)	60 (16–79)	0.382
**Sex, female, *n* (%)**	17 (54.8%)	35 (71.4%)	0.154
**Days from symptom onset to presentation, median (range)**	12 (3–30)	12 (1–45)	0.882
**Symptoms first occurred upon awakening, *n* (%)**	7 (22.6%)	20 (40.8%)	0.145
**Chief presenting symptoms, *n* (%)**			
Rotational vertigo	4 (12.9%)	30 (61.2%)	
Non-rotational vertigo	27 (87.1%)	19 (38.8%)	<0.001
• Swaying/tilting sensation	1 (3.2%)	2 (4.1%)	1.000
• Floating sensation	11 (35.5%)	4 (8.2%)	0.003
• Unsteadiness / imbalance	10 (32.3%)	6 (12.2%)	0.044
• Not firmly grounded while walking	2 (6.5%)	2 (4.1%)	0.639
• Veering to one side	0 (0.0%)	1 (2.0%)	1.000
• Nausea	3 (9.7%)	4 (8.2%)	1.000

Baseline characteristics of patients with suspected BPPV who underwent positional testing. Group 1: DHT(−)/HRST(+); Group 2: DHT(+), irrespective of HRST results.

## Data Availability

The data presented in this study are available upon request from the corresponding author. The data is not publicly available due to privacy restrictions.
